# Time-resolved aperiodic dynamics in event segmentation in attention-deficit/hyperactivity disorder

**DOI:** 10.1093/braincomms/fcag351

**Published:** 2026-09-12

**Authors:** Xianzhen Zhou, Foroogh Ghorbani, Bernhard Hommel, Veit Roessner, Christian Beste, Astrid Prochnow

**Affiliations:** Cognitive Neurophysiology, Department of Child and Adolescent Psychiatry, Faculty of Medicine, TU Dresden, Dresden 01307, Germany; Cognitive Neurophysiology, Department of Child and Adolescent Psychiatry, Faculty of Medicine, TU Dresden, Dresden 01307, Germany; School of Psychology, Shandong Normal University, Jinan 250014, China; Cognitive Neurophysiology, Department of Child and Adolescent Psychiatry, Faculty of Medicine, TU Dresden, Dresden 01307, Germany; German Center for Child and Adolescent Health (DZKJ), Dresden 01307, Germany; Cognitive Neurophysiology, Department of Child and Adolescent Psychiatry, Faculty of Medicine, TU Dresden, Dresden 01307, Germany; German Center for Child and Adolescent Health (DZKJ), Dresden 01307, Germany; Cognitive Neurophysiology, Department of Child and Adolescent Psychiatry, Faculty of Medicine, TU Dresden, Dresden 01307, Germany

**Keywords:** ADHD, event segmentation, aperiodic activity, EEG

## Abstract

Attention-deficit/hyperactivity disorder (ADHD) is associated with difficulties in organizing behaviour over time, yet the neurophysiological mechanisms underlying these temporal control deficits remain poorly understood. The brain continuously segments ongoing experience into discrete events to guide prediction and adaptive action. In our study, we examined whether adolescents with ADHD differ from neurotypical (NT) peers in how they behaviourally and neurally structure continuous experience. Seventy-three adolescents with ADHD and 73 adolescent NT controls viewed a movie while indicating perceived event boundaries. Concurrent EEG recordings were analysed using time-resolved spectral parameterization to track moment-to-moment changes in aperiodic neural activity. Both groups detected meaningful transitions, but NT adolescents segmented more sensitively to situational changes, particularly social and spatial cues. Across the entire recording window, ADHD participants exhibited steeper aperiodic exponents than NT participants across boundary and no-boundary intervals. Time-resolved analyses revealed that, around event boundaries, ADHD participants showed exaggerated pre-boundary steepening and attenuated post-boundary flattening of the aperiodic exponent—signifying over-stabilization before, and insufficient re-engagement after, event transitions. In ADHD only, behavioural segmentation sensitivity correlated with post-boundary aperiodic modulation. Although counterintuitive given common assumptions about increased neural ‘noise’ in ADHD, this pattern indicates that ADHD is characterized by an imbalance between neural stabilization and re-engagement when parsing continuous experience. Steeper aperiodic activity reflects a restricted neural state space that limits adaptive updating, leading to less context-sensitive segmentation. These findings link everyday temporal disorganization in ADHD to alterations in aperiodic neural dynamics and impaired temporal cognition in ADHD.

## Teaser

Zhou et al. report that adolescents with attention-deficit/hyperactivity disorder segment ongoing experience less sensitively than peers. Electroencephalography reveals exaggerated neural stabilization before, and reduced re-engagement after, event transitions. Altered aperiodic brain dynamics restrict adaptive updating, linking everyday temporal disorganization to fundamental neurophysiological mechanisms.

## Graphical Abstract

**Graphical Abstract:**
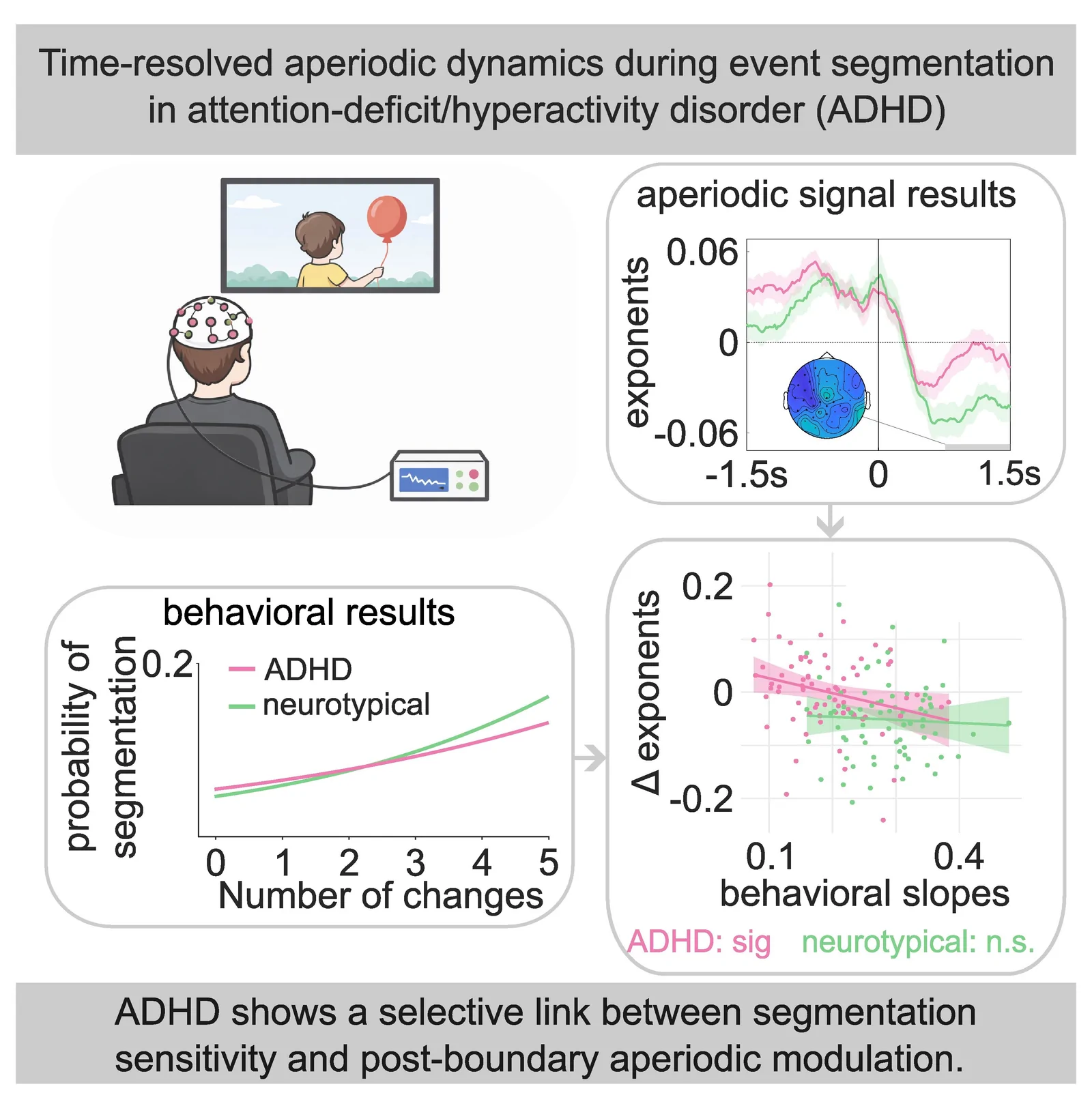

## Introduction

Attention-deficit/hyperactivity disorder (ADHD) is one of the most prevalent neurodevelopmental neuropsychiatric conditions, clinically marked by difficulties in sustaining attention and controlling impulses.^1,2^ Beyond these core diagnostic features, ADHD is often associated with difficulties in organizing goal-directed behaviour over time.^3,4^ Yet, the underlying neurocognitive mechanisms that shape how individuals with ADHD experience and structure the flow of ongoing information remain incompletely understood. Everyday life unfolds as a continuous stream of events that must be parsed into meaningful units for effective prediction and goal-directed behaviour.^5^ Understanding how ADHD affects this temporal organization of experience is essential to bridge the gap between laboratory findings and real-world symptom expression.

Event segmentation theory (EST) outlines how humans may transform the continuous flow of perception into discrete, structured episodes.^5-7^ The EST posits internal models of ‘what is happening now,’ that are updated when sensory input deviates from expectation that are built on the basis of experiences stored as event schemata.^8^ These updates are the basis of event boundaries—moments when one event ends and another begins. Efficient segmentation supports adaptive behaviour in dynamic environments by structuring continuous activity into meaningful units, allowing individuals to update predictions and adjust goals as situations change.^9-11^ Interestingly, ADHD is not only a disorder of attention or inhibition but of temporal control, i.e. the ability to regulate cognitive and neural states across time.^12,13^ Individuals with ADHD often show atypical temporal processing in laboratory timing tasks.^13-15^ Although such tasks differ from event segmentation, prior work has also shown ADHD-related differences in how continuous activity is parsed into meaningful events.^16^ This raises the question of whether atypical neural dynamics during ongoing event perception may contribute to altered event segmentation in ADHD. Neurobiologically, ADHD has been linked to atypical coordination between large-scale networks, including the default mode, salience, and frontoparietal control systems.^17^ Electrophysiological findings converge on reduced flexibility of oscillatory coupling and atypical cross-frequency interactions,^18^ which may reflect an imbalance in the underlying excitation–inhibition (E/I) system.

These abnormalities suggest that ADHD might involve an altered ability to flexibly open and close neural network states in synchrony with environmental change, a process that is central to event segmentation.^6^ If this is the case, one would expect both behavioural segmentation (the detection of situational boundaries) and neural signatures of such transitions to differ from those in typically developing peers. While there is some evidence of altered event segmentation in ADHD,^16^ its neurophysiological basis is only incompletely understood, presumably because, as we argue, only oscillatory (periodic) neural activity has been considered thus far. Interestingly, however, the aforementioned E/I balance is an aspect that affects so-called aperiodic activity, a measure that has recently gained attention as a marker of intrinsic cortical excitability and network balance.^19-22^ On a functional level, mounting evidence indicates that aperiodic activity is important for behavioural adaptation.^23-28^ Intriguingly, recent work suggests a link between event segmentation and processes needed to adapt behaviour.^29^ During behavioural adaptation, information processing can shift along a stability-flexibility continuum.^30-32^ In a stability-dominated mode, information processing is rigid and focused; in a flexibility-dominated mode, information processing is more exploratory, and more information is considered.

Aperiodic activity has been shown to capture changes in cognitive control modes, including shifts between persistence-dominated and flexibility-dominated states,^23-28^ with steeper aperiodic slopes associated with greater persistence. In the context of event segmentation, the period before a perceived boundary may involve maintaining the current event model while accumulating situational changes are evaluated. Thus, pre-boundary steepening of the aperiodic slope may reflect a persistence-dominated processing mode that supports continued maintenance of the current event model while incoming information signalling a potential need for updating is evaluated. After segmentation, flattening of the aperiodic exponent may reflect a shift toward a more flexible processing mode as the prior event model is updated and new information is integrated. If ADHD involves diminished temporal flexibility or impaired regulation of aperiodic activity, one would expect an atypical modulation of the aperiodic exponent around event segmentation. In this study, we examined this hypothesis. Adolescents with and without ADHD watched a continuous 32-min movie while EEG was recorded. Participants were instructed to press a key whenever they perceived that ‘a scene ended and another one began,’ providing an individual measure of event segmentation. EEG data were analysed using a time-resolved spectral parameterization approach to track changes in the aperiodic activity before and after each event boundary. Understanding these mechanisms may help clarify how adolescents with ADHD organize, predict, and adapt to continuous real-world events, while offering a potential link between altered neural dynamics and everyday temporal regulation difficulties in ADHD.

## Materials and methods

### Participants

The study sample comprised *n* = 84 neurotypical (NT) adolescents and *n* = 81 adolescents diagnosed with ADHD, aged 11–16 years. Participants were recruited from an in-house database and through public advertisements. Following an initial telephone screening conducted with the adolescents and/or their legal guardians, eligible individuals were invited to participate. Medicated participants diagnosed with ADHD were included only if they were treated with a methylphenidate-based medication administered as a single morning dose, and their appointments were scheduled for the late afternoon to minimize medication effects. The sample of the current study overlapped with samples used in previous studies.^16,33,34^ Patients with ADHD and NT adolescents as well as their legal guardians provided written informed consent before any study procedure was applied. The study was approved by the local ethics committee of the Medical Faculty of the Technische Universität Dresden and in accordance with the Declaration of Helsinki.

Following data collection, participants were excluded from the final analyses based on EEG-quality, behavioural, and model quality criteria. Poor EEG data quality was defined as having more than 20 interpolated channels after preprocessing, resulting in the exclusion of three NT and two ADHD participants. Participants were also excluded if they produced an insufficient number of behavioural responses, defined as fewer than 10 key presses during the event segmentation task (NT: *n* = 2). Further behavioural exclusions were applied for outlier mean segment lengths, calculated as each participant's average segment length and identified as outliers within each group (NT: *n* = 3; ADHD: *n* = 3). Finally, participants were excluded for poor aperiodic model fits if more than 30% of their model fits had *R*^2^ <0.95 or fitting error >0.05 (NT: *n* = 4; ADHD: *n* = 3). The final sample comprised *n* = 73 NT adolescents (mean age = 13.9 ± 1.7 years; 31 males, 2 diverse; male-to-female ratio = 0.78) and *n* = 73 adolescents with ADHD (mean age = 13.3 ± 1.4 years; 61 males; male-to-female ratio = 5.08). NT participants were slightly older than ADHD participants [*t*(139.41) = 2.13, *P* = 0.035, *d* = 0.353], and the gender distribution differed between groups (χ^2^ = 26.86, *P* < 0.001, φ = 0.429). Prior to the study session, parents completed standardized questionnaires assessing ADHD symptom scores and comorbidities. As expected, parent-reported ADHD symptom scores were significantly higher in the ADHD group; detailed questionnaire results are provided in the Supplementary Material Section S1 and Supplementary Table S1.

### Task

Participants viewed the short film ‘The Red Balloon’, which has a total duration of around 32 min and which was used by previous work from our group.^16,33,35,36^ The film was divided into three clips of approximately 10 min each, with self-paced breaks between clips. During the viewing, participants performed an event segmentation task. They were instructed to ‘press the space key whenever something in the movie ended and something else was about to begin,’ without receiving further guidance regarding the number, length, or content of segments. Each keypress was recorded and served as a behavioural index of event segmentation. Following prior work by Zacks *et al*.^37^, who analysed the movie frame by frame (30 frames per second), nine types of situational changes between consecutive frames were defined (Supplementary Material Section S2). Adopting the rationale of Zacks *et al*.,^37^ the film was segmented into 2-s intervals for the behavioural analyses, resulting in 978 intervals with 60 frames each. For each interval, the number of situational changes was computed, yielding 512 intervals with no situational change, 276 with one, 104 with two, 46 with three, 30 with four, and 10 with five or more situational changes.

### EEG recording and preprocessing

EEG data were recorded using elastic caps (EasyCap Inc.) equipped with 60 Ag/AgCl electrodes, referenced to Fpz and grounded at θ = 58, φ = 78. Signals were amplified with BrainAmp amplifiers (Brain Products Inc.), and electrode impedances were kept below 5 kΩ. Data were acquired at a sampling rate of 500 Hz and subsequently down-sampled to 300 Hz during offline preprocessing. Preprocessing was performed using the ‘Automagic’ pipeline^38^ and EEGLAB.^39^ Initially, flat channels were identified and removed, followed by re-referencing the data to the average reference. The PREP and EEGLAB clean_rawdata() pipelines were then applied. Line noise at 50 Hz was removed, and a robust average reference was applied after eliminating contaminated channels. A finite impulse response (FIR) high-pass filter (0.5 Hz; order = 1286; stop-band attenuation = 80 dB; transition band = 0.25–0.75 Hz) was used to detect and remove flat-lined, noisy, or outlier channels. To suppress EMG activity, a low-pass sinc FIR filter at 100 Hz (order = 86)^40,41^ was applied. Electro-oculographic artefacts were corrected using the subtraction approach described by Parra *et al*.^42^ Subsequently, Independent Component Analysis combined with the Multiple Artifact Rejection Algorithm (MARA)^43^ was employed to identify and remove residual muscle, cardiac, and ocular artefacts. Artefact subspace reconstruction (burst criterion = 15)^44^ was used to reconstruct epochs exhibiting abnormally high power (>15 SD relative to calibration data). Time windows that could not be reconstructed were excluded. Missing or removed channels were interpolated using spherical interpolation. Further analyses were conducted in FieldTrip.^45^

**Figure 1:**
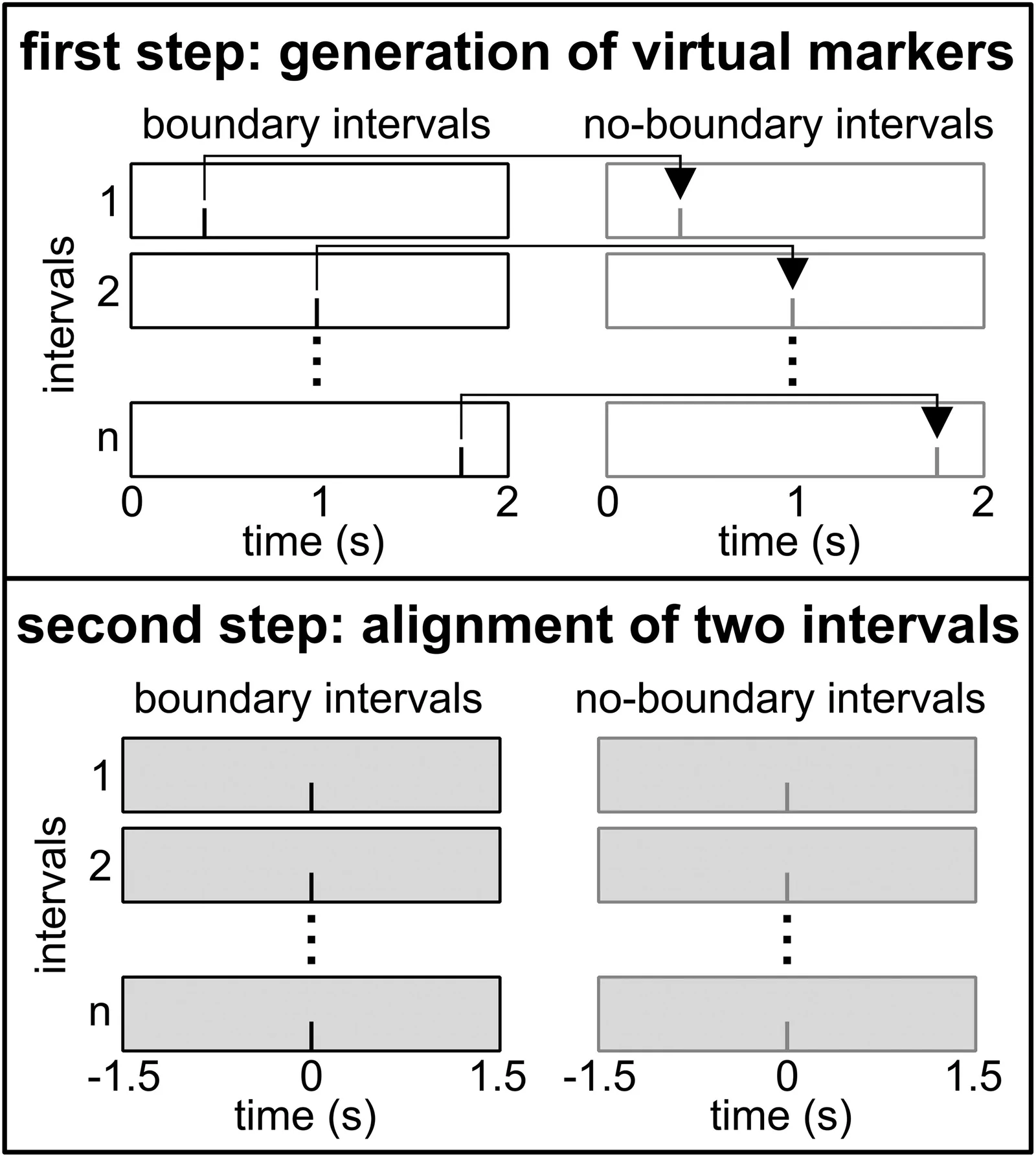
**Schematic illustration of the creation of virtual markers for no-boundary intervals.** Boundary intervals (black) and no-boundary intervals (grey) are shown. In the first step, continuous EEG data were segmented into 2-s intervals, aligned with the behavioural data. Each boundary interval was randomly paired with a no-boundary interval. Virtual markers were then placed within the no-boundary intervals at the same relative time point as the key press in the corresponding boundary interval. In the second step, EEG data were re-segmented based on these virtual markers, enabling the analysis of activity from −1.5 to +1.5 s relative to each marker.

To distinguish between time periods containing event boundaries and those without, data were segmented into boundary intervals (BI), corresponding to key presses marking event boundaries, and no-boundary intervals (NBI), segments without such key presses, following the approach of Prochnow *et al*.^35^ Because BI inherently contained behavioural response markers, while NBI did not, virtual markers were generated for NBI to match the temporal characteristics of BI through the procedure shown in Fig. 1. For subsequent analyses, EEG data from −1.5 s to +1.5 s relative to each (real or virtual) marker were extracted and used.

### Time-resolved aperiodic signal analysis

To derive the time-resolved aperiodic signal, we computed sliding-window power spectra for each trial using a multitaper approach implemented in FieldTrip,^45^ with 500-ms windows and a 20-ms time step.^46,47^ This procedure yielded power estimates for 73 frequency bins spanning 4–40 Hz, enabling the estimation of instantaneous spectral parameters. We then applied spectral parameterization to estimate the aperiodic component of the power spectrum at each time point and channel. The parameters were configured as follows in the current study^46^: peak width limits = (2, 8), maximum number of peaks = 4, minimum peak height = 0, peak threshold = 2.0, and aperiodic mode = ‘fixed’. Power spectra were parameterized within the 4–40 Hz frequency range, and the instantaneous aperiodic exponents were extracted from the fitted spectra for each time point and channel. Additionally, *R*^2^ and error values were obtained to assess the goodness of fit of the model. A detailed description of the spectral-parameterization model formulation is provided in the Supplementary Material Section S3.

### Statistical analyses

The experimental unit was the individual participant. Behavioural effects were analysed using generalized linear mixed-effects logistic regression model implemented using the ‘glmer’ function in R. In the first analysis, the movie was divided into 2-s time bins. The predictor variable was the number of situational changes within each interval (ranging from 0 to 5), while the dependent variable indicated whether a response occurred during that interval (0 = no response, 1 = response). The model included number of situational changes, group and their interaction as fixed effects. To account for inter-individual variability in baseline response probability, random intercepts were included for each participant. The first mixed-effects model was specified as: response ∼ number of changes * group + (1 | participant). In the second analysis, the predictors were group, the occurrence of each specific type of situational change within an interval (0 = no occurrence, 1 = occurrence), and their interactions. The dependent variable was whether one or more responses occurred in the interval. The second mixed-effects model was specified as: response ∼ change type * group + (1 | participant), where change type comprised nine binary predictors as described in Task part. Random intercepts were again included for participants. The models were fitted assuming a binomial error distribution with a logit link function. Odds ratios (ORs) were derived from the fixed-effect coefficients, and multicollinearity was assessed using the variance inflation factor (VIF).

To examine temporal and spatial differences in the aperiodic exponent across conditions and groups, cluster-based permutation tests (CBPTs) were performed using FieldTrip,^45^ with clusters defined using a sample-level threshold (α = 0.05), cluster-level statistics computed as the sum of t-values, and significance assessed against a null distribution generated from 1000 random permutations. To ensure data quality, the goodness-of-fit was checked prior to statistical analysis to exclude participants with poor aperiodic model fits. Only participants with at least 70% of channels meeting the following criteria were retained for further analysis: *R*^2^ > 0.95 and error <0.05. Finally, we conducted a regression analysis (‘lm’ function in R) to assess whether individual behavioural sensitivity to situational changes was associated with aperiodic neural dynamics. Behavioural sensitivity, referred to as the behavioural slope, was defined as each participant's regression coefficient for the association between the number of situational changes and segmentation probability.

## Results

### Behaviour

**Figure 2:**
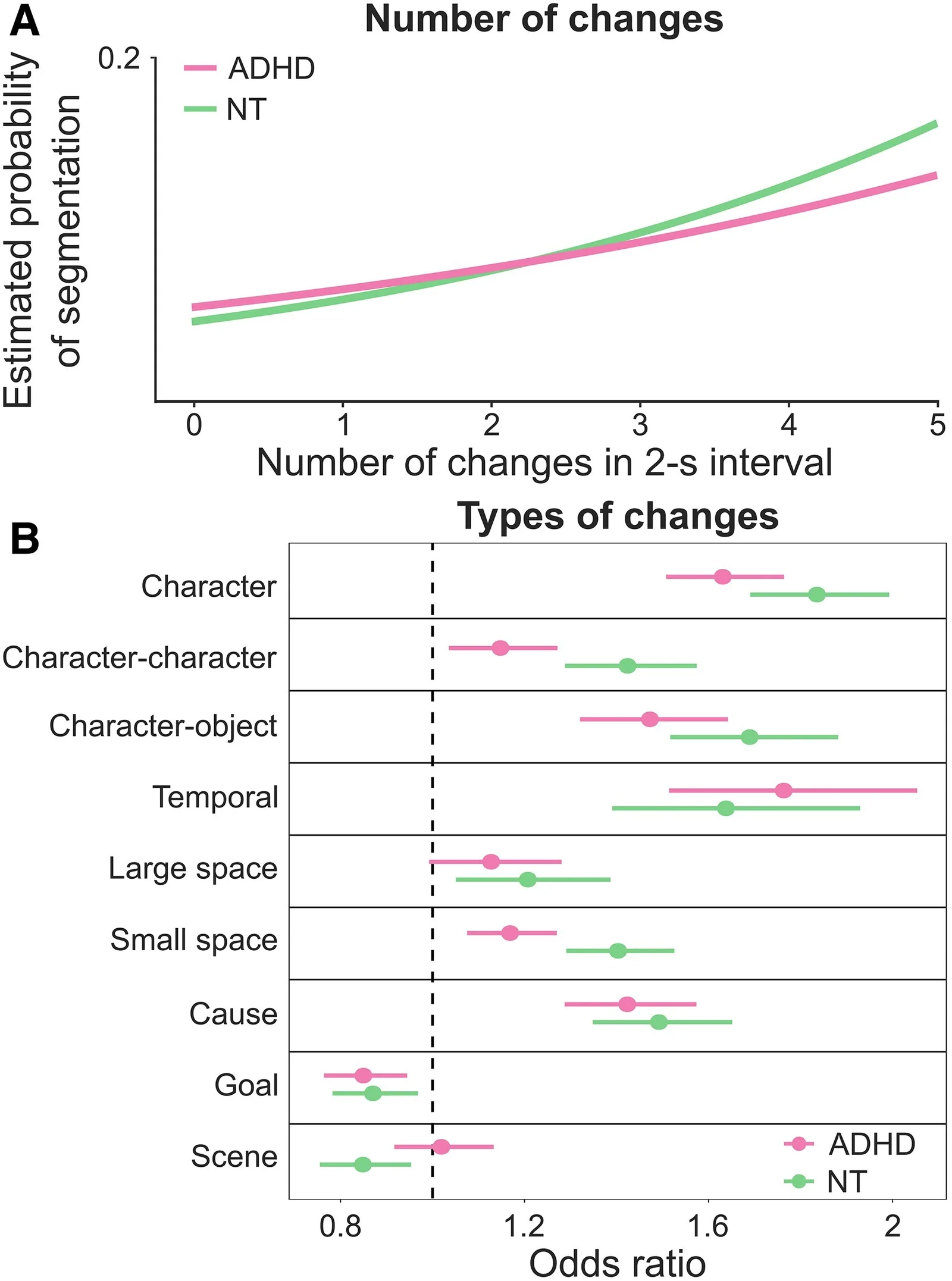
**Behavioural segmentation performance during movie viewing.** (**A**) Estimated probability of segmentation as a function of the number of situational changes within each 2-s interval for the attention-deficit/hyperactivity disorder (ADHD) (pink) and neurotypical (NT, green) groups. Mixed-effects logistic regression showed a significant main effect of number of changes, β = 0.31, *P* < 0.001, and a significant Number of changes × Group interaction, β = −0.09, *P* = 0.001. Both groups showed increasing segmentation probability with more situational changes, but the NT group exhibited a steeper slope, indicating a stronger sensitivity of change density on boundary detection. (**B**) Odds ratios (±95% confidence interval) from the mixed-effects logistic regression for individual types of situational changes. Full statistics for the model are reported in Table 1. Values greater than 1 indicate an increased likelihood of boundary placement relative to baseline. NT participants showed stronger sensitivity to several change types, particularly character-related and spatial (small and large space) changes, whereas ADHD participants displayed a generally attenuated pattern. In both analyses, the experimental unit was the participant, and interval-level segmentation responses were analysed using mixed-effects logistic regression with participant-level random effects.

The results of the mixed-effects logistic regression analyses are presented in Fig. 2.

The first model predicting segmentation behaviour from the number of situational changes per interval and group membership (intercept: −2.96, *P* < 0.001; OR = 0.05; 95% CI [0.04, 0.06]; VIF ≤ 2.02; Fig. 2A) revealed significant main effects of the number of situational changes (β = 0.31, *P* < 0.001; OR = 1.36; 95% CI [1.33, 1.40]) and, importantly, a significant interaction between situational changes and group membership (β = −0.09, *P* = 0.001; OR = 0.91; 95% CI [0.88, 0.94]). The probability of segmentation was more strongly influenced by the number of situational changes in the NT group (intercept: −3.20, *P* < 0.001; β = 0.31, *P* < 0.001; OR = 1.36; 95% CI [1.33, 1.40]) than in the ADHD group (intercept: −2.99, *P* < 0.001; β = 0.22, *P* < 0.001; OR = 1.24; 95% CI [1.21, 1.27]). However, group membership alone did not significantly predict segmentation probability (β = 0.14, *P* = 0.27; OR = 1.15; 95% CI [0.90, 1.47]). Because age and gender differed between groups, we conducted an additional control analysis including age and gender as covariates. This analysis yielded the same pattern of results and is reported in the Supplementary Material Section S4.

A second mixed-effects logistic regression model (Fig. 2B; Table 1), examining segmentation behaviour as a function of the type of situational change and group membership, identified significant interactions for character changes, character–character interaction changes, small space change and scene changes. Specifically, character changes were more predictive of segmentation behaviour in the NT group than in the ADHD group, as well as character–character interaction changes and small space changes. Moreover, in the NT group, large space changes and scene change significantly predicted segmentation, whereas this effect was non-significant in the ADHD group (Supplementary Material Section S5 containing Supplementary Tables S2 and S3). No significant interactions between group membership and any other types of situational changes were observed.

**Table 1:** Mixed-effects logistic regression predicting segmentation responses from types of situational changes and group

Predictor	β	*z*	*P*	OR	95% CI for OR
Intercept	−1.987	−28.522	**<0.001**	0.137	[0.120, 0.157]
Character	0.548	18.922	**<0.001**	1.730	[1.635, 1.831]
Character–character	0.246	6.707	**<0.001**	1.279	[1.190, 1.374]
Character–object	0.456	11.675	**<0.001**	1.577	[1.461, 1.703]
Temporal	0.530	9.314	**<0.001**	1.700	[1.520, 1.900]
Large space	0.154	3.210	0.**001**	1.167	[1.062, 1.282]
Small space	0.247	8.212	**<0.001**	1.281	[1.207, 1.359]
Cause	0.377	10.333	**<0.001**	1.457	[1.357, 1.565]
Goal	−0.151	−3.934	**<0.001**	0.860	[0.798, 0.927]
Scene	−0.073	−1.809	0.071	0.930	[0.860, 1.006]
Group	0.037	0.536	0.592	1.038	[0.906, 1.190]
Character × Group	0.059	2.039	**0.041**	1.061	[1.002, 1.123]
Character–character × Group	0.108	2.948	**0.003**	1.114	[1.037, 1.197]
Character–object × Group	0.069	1.759	0.079	1.071	[0.992, 1.156]
Temporal × Group	−0.037	−0.648	0.517	0.964	[0.862, 1.078]
Large space × Group	0.034	0.712	0.476	1.035	[0.942, 1.137]
Small space × Group	0.091	3.037	**0.002**	1.096	[1.033, 1.162]
Cause × Group	0.024	0.649	0.517	1.024	[0.953, 1.100]
Goal × Group	0.012	0.315	0.753	1.012	[0.939, 1.091]
Scene × Group	−0.092	−2.285	**0.022**	0.912	[0.844, 0.987]

Results are from a generalized linear mixed-effects logistic regression predicting segmentation response. Participant was included as a random intercept. OR = odds ratio; CI = confidence interval. Significant *P*-values are highlighted in bold.

### Aperiodic activity

**Figure 3:**
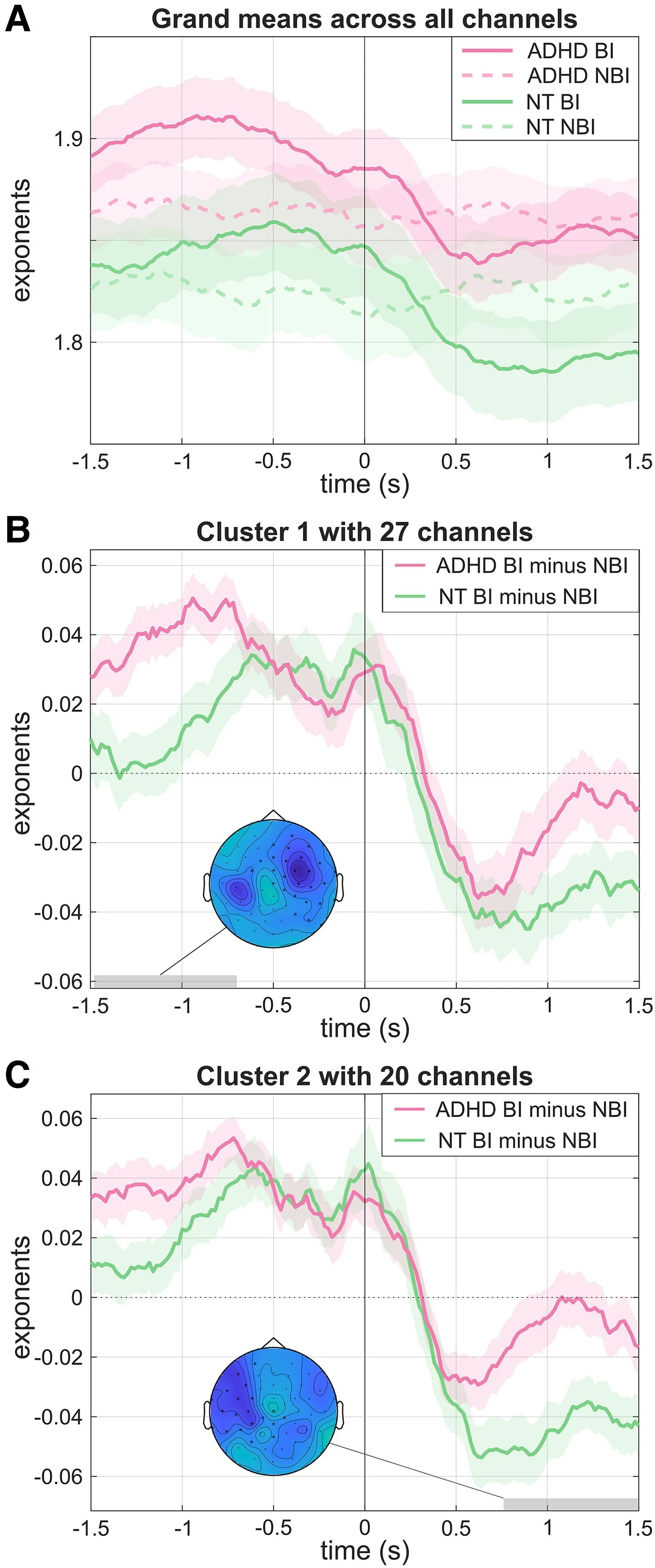
**Neural differentiation in the aperiodic exponent.** (**A**) Grand-average exponent time courses (mean ± SEM) for boundary intervals (BIs) and non-boundary intervals (NBIs) in the attention-deficit/hyperactivity disorder (ADHD) (pink) and neurotypical (NT, green) groups. The exponent reflects the aperiodic slope of the EEG power spectrum, with lower values indicating relatively flatter spectral profiles. (**B–C**) Cluster-based permutation tests (CBPTs) identified two significant negative clusters showing a Group × Condition interaction. Cluster 1 occurred before segmentation, from −1.48 to −0.70 s, *P* = 0.031, and BI-related steepening that was more pronounced in the ADHD group compared to NT group. Cluster 2 emerged after segmentation, from 0.76 to 1.50 s, *P* = 0.034, and showed stronger BI-related flattening in the NT group compared to ADHD group. Shaded regions represent ± SEM, grey bars along the x-axis mark significant time windows of the Group × Condition interaction clusters identified by CBPT, and scalp insets indicate electrodes contributing to each cluster. The experimental unit was the participant; CBPT was performed on participant-level across electrodes and time points.

To assess group- and condition-related differences in the time-resolved aperiodic exponent, we first averaged the exponent values across all electrodes for each participant, providing an overview of temporal fluctuations as shown in Fig. 3A. This analysis allowed visualization of overall trends in aperiodic activity across the two groups and two conditions over time.

Further, to statistically assess spatiotemporal differences, we conducted CBPTs across all electrodes and time points within the full −1.5 to +1.5 s analysis window. These analyses evaluated main effects of condition (BI versus NBI), main effects of group (NT versus ADHD), and the Group × Condition interaction, thereby identifying significant clusters that reflected reliable differences in the aperiodic exponent across time and space. The CBPT revealed significant effects for both condition and group, as well as their interaction as shown in Fig. 3B and C. For the condition effect, calculated as BI minus NBI, the NT group showed a positive cluster (*P* < 0.001) from −1.0 to 0.28 s, indicating a steeper aperiodic exponent in BI than NBI before the boundary, and a negative cluster (*P* < 0.001) from 0.36 to 1.5 s, indicating a flatter exponent in BI than NBI after the boundary. The ADHD group showed a similar pattern, with a positive cluster (*P* < 0.001) from −1.5 to 0.26 s and a negative cluster (*P* = 0.016) from 0.4 to 0.94 s. These effects were most prominent over central and parietal regions, suggesting differential modulation of the aperiodic exponent around event boundaries. For the group effect, calculated as NT minus ADHD, significant negative clusters were observed across nearly the entire analysis window (−1.5 to 1.5 s) in both BI (*P* = 0.020) and NBI (*P* = 0.037) conditions. These negative clusters indicate a flatter aperiodic exponent in the NT group than in the ADHD group, with the strongest effects over frontocentral and parietal electrodes. Notably, the Group × Condition interaction revealed two significant negative clusters: the first from −1.48 to −0.7 s (*P* = 0.031) before segmentation, and the second from 0.76 to 1.5 s (*P* = 0.034) after segmentation. These interaction effects indicate group differences in condition-related (BI – NBI) modulation of the aperiodic exponent, with different degrees of BI-related steepening before segmentation and BI-related flattening after segmentation. Topographical maps illustrate that these effects were most prominent over frontocentral and parietal regions.

### Regression analysis

**Figure 4:**
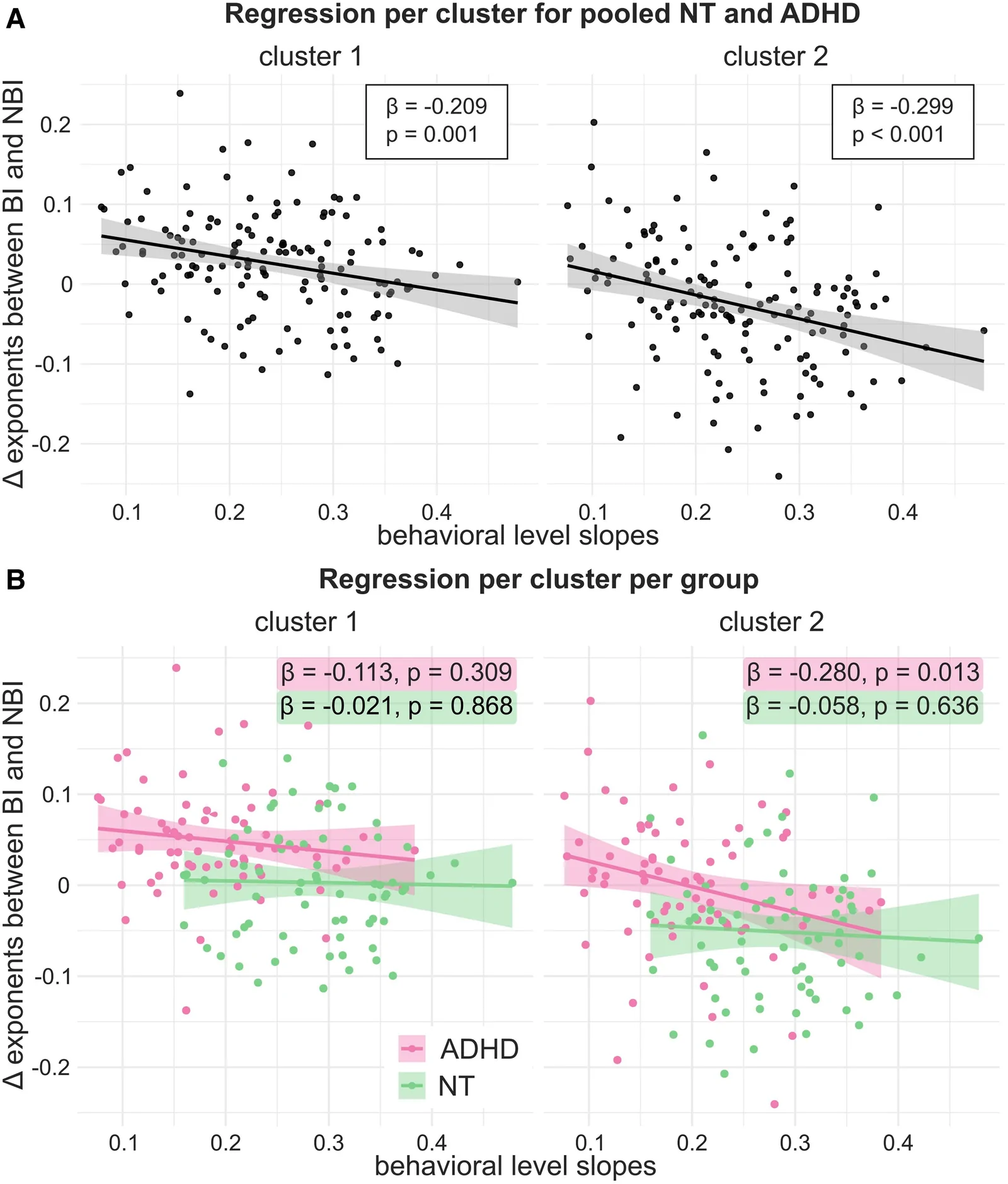
**Relationship between behavioural segmentation sensitivity and neural aperiodic changes.** (**A**) Across all participants in two groups, regression analyses linked the behavioural slopes from the mixed-effects model (x-axis) to the neural Δ exponent (BI − NBI) per cluster (y-axis). Significant negative associations were found for Cluster 1, β = −0.209, *P* = 0.001, and Cluster 2, β = −0.299, *P* < 0.001. Negative β values indicate that individuals with stronger behavioural segmentation sensitivity showed larger neural differentiation between boundary and non-boundary intervals. (**B**) Separate regressions for the attention-deficit/hyperactivity disorder (ADHD) (pink) and neurotypical (NT) (green) groups. A significant negative association was only found in ADHD group for Cluster 2, β = −0.280, *P* = 0.013, indicating that stronger behavioural segmentation sensitivity was linked to larger neural differentiation between conditions after segmentation. Shaded bands = 95% confidence intervals; β = standardized regression coefficient. The experimental unit was the participant.

To further examine the relationship between neural and behavioural measures, we extracted the mean aperiodic exponent within each of the two significant clusters identified in the Group × Condition interaction and performed regression analyses with individual behavioural slopes derived from the logistic regression model. Across all participants, both clusters showed significant negative relationships between behavioural slopes and the exponent difference (BI − NBI, Fig. 4A; Cluster 1: β = −0.209, *P* = 0.001; Cluster 2: β = −0.299, *P* < 0.001). This indicates that individuals who exhibited greater sensitivity to situational changes—that is, who segmented more frequently as the number of situational changes increased—also showed a stronger reduction in the aperiodic exponent difference between conditions.

When examined separately by group, a significant negative correlation was observed only in the post-segmentation cluster for the ADHD group (Fig. 4B; β = −0.280, *P* = 0.013), whereas no significant associations were found in the NT group (pre-segmentation cluster: β = 0.021, *P* = 0.868; post-segmentation cluster: β = −0.058, *P* = 0.636). These findings indicate that, in individuals with ADHD, greater behavioural sensitivity to event segmentation is linked to stronger modulation of the aperiodic exponent during time windows following segmentation, that is, at the onset of a new event.

## Discussion

This study investigated how adolescents with and without ADHD segment continuous naturalistic experiences and how these behavioural processes relate to time-resolved aperiodic EEG dynamics. Using a movie-viewing paradigm, we observed that event segmentation was associated with systematic modulations in the aperiodic component of neural activity, but that the temporal pattern and overall level of this modulation differed markedly between groups.

At the behavioural level, both groups detected meaningful transitions in the movie, but NT adolescents adjusted their segmentation behaviour more sensitively to situational changes. This heightened sensitivity to situational changes is evident in NT adolescents’ utilization of a broad range of information, particularly social and spatial information, such as changes in character and interactions. In contrast, individuals with ADHD were less likely to identify an event segment when faced with the same amount of information, suggesting that they use a smaller set of informational cues. This pattern may suggest a less differentiated mapping between external situational dynamics and internal event representations in ADHD, consistent with prior evidence of reduced contextual updating in the disorder.^48^ However, the results from the aperiodic activity analysis rather support the interpretation that ADHD participants seem to monitor and rely on fewer information ‘channels’ or ‘features’^49^ to interpret ongoing events,^16^ indicating reduced dimensionality of the considered information.

Adolescents with ADHD showed steeper aperiodic slopes than NT individuals across the entire analysis window. At first glance, this result may appear counterintuitive, since ADHD is often associated with increased neural variability or ‘noisiness’.^24,50,51^ One might therefore expect flatter, not steeper, aperiodic slopes. This is even more the case since flatter aperiodic exponents have been associated with more flexible cognitive control modes^23-28^ that may also be characteristic of ADHD. However, this apparent paradox becomes meaningful when considering what the aperiodic exponent may reflect neurophysiologically. A steeper slope corresponds to a neural system dominated by low-frequency, large-scale activity patterns^19^ and thus processes also associated with more rigid network coupling.^52^ Hence, rather than reflecting ‘less noise’ in a beneficial sense, the steeper slope in ADHD likely indicates a diminished ability to reconfigure processing resources dynamically. In this view, ADHD neural processes may operate in a globally more stable but less adaptive regime.^32^ In this less adaptive regime, fewer network states may be accessible, leaving fewer degrees of freedom to process different features of incoming information.^53^

Most importantly, time-resolved analyses further demonstrated that these group differences are not static but context-dependent. In both groups, the aperiodic exponent steepened before an event boundary, consistent with transient inhibitory stabilization, and flattened afterward, consistent with re-engagement and excitatory resetting as a new event unfolds. However, the magnitude and duration of these modulations differed systematically between groups. Before event boundaries, ADHD participants exhibited a stronger pre-boundary steepening, suggesting excessive inhibitory ‘closure’ or an over-stabilization of the current neural state. After boundaries, their post-boundary flattening was smaller and shorter-lived, pointing to a weaker or less sustained re-engagement. In contrast, NT adolescents displayed a well-balanced pattern, an oscillatory-like alternation supporting continuous event updating. Thus, ADHD appears to involve a temporal imbalance: an over-controlled ‘braking’ response before event transitions, followed by insufficient release of it and shift toward more flexibility-related network states. Such a pattern echoes meta-control models of ADHD that propose difficulty in flexibly shifting between cognitive states.^54^

Importantly, in ADHD, neural modulation of the aperiodic exponent correlates with behavioural segmentation sensitivity, but only during the post-segmentation window. This finding suggests that, in adolescents with ADHD, sensitivity to situational changes during event segmentation may be more related to how effectively the brain re-engages after an event closure, i.e. how effectively it shifts towards different information-processing state after segmentation. This interpretation fits with the behavioural results: ADHD participants were not globally less likely to segment, but their segmentation responses were less strongly guided by several contextual changes. In NT adolescents, behaviour was less tightly coupled to these neural dynamics, implying a more autonomous and resilient segmentation mechanism. Together, these findings suggest that, in ADHD, reduced sensitivity to contextual changes may be related to weaker or less sustained post-segmentation neural re-engagement. Their networks may rely on fewer representational features when interpreting complex, dynamic environments, resulting in less differentiated internal models of ongoing events. However, because aperiodic activity reflects a broad stability–flexibility balance influenced by multiple neural processes rather than a single neural mechanism,^19^ interpretations linking aperiodic modulation to specific inhibitory or excitatory control states should be regarded as inferential.

These findings should also be interpreted in light of the task design. BIs necessarily included a button press, whereas NBIs did not. Although the main aperiodic effects extended beyond the immediate response period and the two groups did not differ in total response number, response-related decision or motor activity cannot be fully excluded. In addition, because the analysis was restricted to the −1.5 to +1.5 s window around segmentation, we cannot determine whether activity further before event boundaries resembled NBIs. Future studies using delayed reports, response-free designs, or longer analysis windows could help disentangle event-boundary processing from response-related activity and provide a more complete characterization of neural dynamics before and after perceived boundaries.

In summary, ADHD is marked by an altered neural balance between stabilization and re-engagement during the segmentation of continuous experience. Although adolescents with ADHD show steeper aperiodic slopes, this pattern should not be interpreted as improved neural efficiency; rather, it may reflect reduced flexibility and a lower dimensionality of processing. Their neural systems appear more constrained, relying on fewer features to interpret events, leading to less adaptive segmentation of experience. These findings link everyday temporal disorganization in ADHD to fundamental alterations in aperiodic neural activity.

## Supplementary Material

fcag351_Supplementary_Data

## Data Availability

The data are not publicly available due to ethical regulation. The data that support the findings of this study are available from the corresponding author upon reasonable request. The analysis code used for the behavioural and EEG analyses is available on OSF: https://osf.io/wexfp/overview?view_only=8d4bb5053689428896a947efc633ad5d.
