# Supplementary material for: Time-resolved aperiodic dynamics in event segmentation in attention-deficit/hyperactivity disorder
Source: Brain Commun. 2026 Sep 12;8(5):fcag351. doi: 10.1093/braincomms/fcag351 (PMC13590235; doi:10.1093/braincomms/fcag351)
Supplement: Supplementary file 1 [file fcag351_supplementary_data.docx]

**Supplementary Information**

**Time-resolved aperiodic dynamics in event segmentation in attention-deficit/hyperactivity disorder**

X. Zhou, F. Ghorbani, B. Hommel, V. Roessner, C. Beste, A. Prochnow

**S1. Parent reported questionnaires**

Before the study session, the parents of the participants completed an online questionnaire via SoSci survey^3^, assessing comorbidities and ADHD symptomatology using the Conners Short Form^4^ and the ADHD Questionnaire for Parents (‘Fremdbeurteilungsbogen ADHS’, FBB-ADHS^1^). The questionnaire was completed for n = 70 NT participants and n = 64 ADHD participants. As expected, ADHD symptom scores were significantly higher in the ADHD group compared with the NT group (see Suppl. Tab. 1 for details).

According to clinical records, n = 29 participants in the ADHD group had at least one additional diagnosis. Moreover, parents reported regular medication for ADHD-related symptoms for n = 30 participants. All participants additionally completed the IQ screening of the Intelligence and Development Scales–2 (IDS-2)^2^ during their study appointment, consisting of the subtests Matrices Completion and Naming Categories. NT participants showed better performance in the IQ screening compared to the ADHD patients, mainly due to better performance in the subtest Naming Categories, while the difference in the subtest Matrices Completion was not significant. Importantly, both groups were in the average IQ range.

*Supplementary Table S1 – Comparison of questionnaire data of parent’s questionnaires between NT and ADHD groups.*

| **Scale** | **NT: M ± SD** | **ADHD: M ± SD** | **\|t\|** | **df** | **p** | **d** |
| --- | --- | --- | --- | --- | --- | --- |
| Conners: inattention | 51.7 ± 8.9 | 65.2 ± 7.5 | 9.51 | 132 | < 0.001 | -1.645 |
| Conners: hyperactivity / impulsivity | 49.9 ± 5.8 | 61.7 ± 8.5 | 9.27 | 110.0 | < 0.001 | -1.630 |
| Conners: learning problems | 47.2 ± 7.7 | 57.0 ± 10.3 | 6.16 | 116.5 | < 0.001 | -1.079 |
| Conners: executive functioning | 50.7 ± 8.7 | 63.5 ± 7.0 | 9.30 | 132 | < 0.001 | -1.609 |
| Conners: aggression | 53.0 ± 6.6 | 58.2 ± 9.2 | 3.71 | 113.7 | < 0.001 | -0.651 |
| Conners: peer relations | 53.9 ± 7.5 | 61.0 ± 7.9 | 5.33 | 132 | < 0.001 | -0.922 |
| FBB-ADHS: inattention | 0.46 ± 0.46 | 1.72 ± .66 | 12.75 | 111.3 | < 0.001 | -2.239 |
| FBB-ADHS: hyperactivity | 0.05 ± 0.12 | 0.74 ± 0.62 | 8.68 | 67.54 | < 0.001 | -1.565 |
| FBB-ADHS: impulsivity | 0.19 ± 0.30 | 1.20 ± 0.83 | 9.16 | 77.70 | < 0.001 | -1.640 |
| FBB-ADHS: total score | 0.26 ± 0.24 | 1.27 ± 0.57 | 13.19 | 83.20 | < 0.001 | -2.355 |
| FBB-ADHS: competencies | 1.74 ± 0.68 | 0.73 ± 0.59 | 9.13 | 132 | < 0.001 | 1.579 |
| IDS-2: IQ Screening | 102.6 ± 13.4 | 95.6 ± 20.1 | 2.48 | 125.6 | 0.014 | 0.411 |
| IDS-2: Naming Categories | 111.4 ± 12.7 | 103.9 ± 21.8 | 2.55 | 115.6 | 0.012 | 0.422 |
| IDS-2: Matrices Completion | 97.3 ± 14.4 | 92.8 ± 17.7 | 1.67 | 144 | 0.098 | - |

*Note.* ADHD – ADHD patient group; FBB-ADHS - ADHD Questionnaire for Parents (‘Fremdbeurteilungsbogen ADHS’); M – mean; NT – neurotypical group; SD – standard deviation. Degrees of freedom have been corrected if equal variances could not be assumed.

**S2. Description of situational-change categories.**

The nine situational-change categories were defined as follows. First, character changes were coded when the focus of the ongoing action shifted from one animate character, or group of characters, to another. Second, character–character interaction changes referred to changes in interactions between characters, including physical or abstract interactions such as touching, conversing, gesturing, approaching one another, or moving together while walking or running. Third, character–object changes were coded when a character’s interaction with an object changed, for example when a character picked up an object, put it down, or began using an object in a new way. Fourth, temporal changes were coded when the current frame was temporally discontinuous from the preceding frame. Fifth, large spatial changes referred to changes in the character’s broader location from one scene to the next. Sixth, small spatial changes referred to changes in the narrative point of view or shot scale, such as changes in how the same location or action was visually framed. Seventh, cause changes were coded when the activity shown in the current frame could not be causally explained by information visible in the immediately preceding frame. Eighth, goal changes were coded when a character’s action was associated with a goal or intention that differed from the one inferred in the preceding frame. Finally, scene changes referred to cuts in which one shot changed to a new shot.

**S3. Spectral-parameterization model description.**

Spectral parameterization was used to decompose the power spectrum into periodic and aperiodic components. The aperiodic component was modeled on the semi-log power spectrum, where frequency values are represented linearly and power values logarithmically. In this formulation, the parameters b, k, and χ define the aperiodic component as:

𝐿(𝐹) = 𝑏 − log (𝑘 + 𝐹^𝜒^)

Here, 𝑏 denotes the broadband offset, 𝑘 represents the knee parameter, 𝜒 is the exponent describing the slope of the aperiodic fit, and 𝐹 is the array of frequency values. The knee parameter is optional; when included, it introduces a bend in the aperiodic 1/f-like component, whereas setting 𝑘 = 0 results in a straight 1/f relationship. In the present study, the aperiodic component was fit in fixed mode, corresponding to k=0. Power spectra were parameterized within the 4–40 Hz range, and the resulting aperiodic exponents were extracted for each channel and time point.

**S4. Control analysis including age and gender**

To assess whether demographic differences between groups influenced the segmentation-related behavioural effects, we fitted an additional generalized linear mixed-effects logistic regression model (‘glmer’ function in R) including age and gender as covariates:

Response ∼ Num_of_Change * Group + Age + Gender + (1∣Subject)

A random intercept for subject was included to account for inter-individual variability in baseline response probability (random intercept variance = 0.547, SD = 0.740). Briefly, the number of situational changes was a strong positive predictor of response probability (β = 0.309, SE = 0.012, z = 25.73, p < .001). Crucially, the interaction between situational change frequency and group remained significant after controlling for age and gender (β = −0.094, SE = 0.017, z = −5.56, p < .001), indicating a reduced sensitivity to situational changes in the ADHD group. Neither age (β = 0.005, SE = 0.042, z = 0.12, p = .901) nor gender showed a significant main effect on response probability (male vs. female: β = 0.088, SE = 0.145, z = 0.61, p = .544; diverse vs. female: β = 0.382, SE = 0.548, z = 0.70, p = .486). The main effect of group was also not significant (β = 0.115, SE = 0.139, z = 0.83, p = .409).

**S5. Post-hoc mixed-effects logistic regressions**

*Supplementary Table S2 Post-hoc mixed-effects logistic regressions predicting segmentation responses from types of situational changes in the ADHD group*

| **Predictor** | **β** | **z** | **p** | **OR** | **95% CI for OR** |
| --- | --- | --- | --- | --- | --- |
| Intercept | -3.060 | -31.908 | **< .001** | 0.047 | [0.039, 0.057] |
| Character | 0.489 | 12.161 | **< .001** | 1.631 | [1.508, 1.765] |
| Character-character | 0.138 | 2.617 | **.009** | 1.148 | [1.035, 1.272] |
| Character-object | 0.387 | 6.943 | **< .001** | 1.473 | [1.320, 1.643] |
| Temporal | 0.567 | 7.248 | **< .001** | 1.764 | [1.513, 2.056] |
| Large space | 0.120 | 1.835 | .066 | 1.127 | [0.992, 1.282] |
| Small space | 0.156 | 3.653 | **< .001** | 1.169 | [1.075, 1.271] |
| Cause | 0.353 | 6.855 | **< .001** | 1.424 | [1.287, 1.575] |
| Goal | -0.163 | -2.995 | **.003** | 0.850 | [0.764, 0.945] |
| Scene | 0.019 | 0.352 | .725 | 1.019 | [0.917, 1.134] |

*Note.* Results are from group-specific generalized linear mixed-effects logistic regression models predicting segmentation response. Participant was included as a random intercept. OR = odds ratio; CI = confidence interval. Significant p-values are highlighted in bold.

*Supplementary Table S3 Post-hoc mixed-effects logistic regressions predicting segmentation responses from types of situational changes in the neurotypical group*

| **Predictor** | **β** | **z** | **p** | **OR** | **95% CI for OR** |
| --- | --- | --- | --- | --- | --- |
| Intercept | -3.249 | -38.217 | **< .001** | 0.039 | [0.033, 0.046] |
| Character | 0.607 | 14.432 | **< .001** | 1.835 | [1.690, 1.993] |
| Character-character | 0.353 | 6.874 | **< .001** | 1.424 | [1.287, 1.575] |
| Character-object | 0.524 | 9.496 | **< .001** | 1.689 | [1.516, 1.882] |
| Temporal | 0.493 | 5.873 | **< .001** | 1.638 | [1.389, 1.931] |
| Large space | 0.188 | 2.642 | **.008** | 1.207 | [1.050, 1.388] |
| Small space | 0.339 | 7.911 | **< .001** | 1.403 | [1.290, 1.526] |
| Cause | 0.400 | 7.694 | **< .001** | 1.492 | [1.347, 1.652] |
| Goal | -0.139 | -2.546 | **.011** | 0.871 | [0.782, 0.969] |
| Scene | -0.164 | -2.747 | **.006** | 0.849 | [0.755, 0.954] |

*Note.* Results are from group-specific generalized linear mixed-effects logistic regression models predicting segmentation response. Participant was included as a random intercept. OR = odds ratio; CI = confidence interval. Significant p-values are highlighted in bold.

**Supplementary References:**

1.	Döpfner M, Görtz-Dorten A. *DISYPS-III Manual : Diagnostik-System Für Psychische Störungen Nach ICD-10 Und DSM-5 Für Kinder Und Jugendliche - III*. 2., erweiterter Nachdruck 2019 der 1. Auflage 2017. Hogrefe; 2019.

2.	Grob A, Hagmann-von Arx P. *IDS-2: Intelligenz- Und Entwicklungsskalen Für Kinder Und Jugendliche*. Hogrefe; 2018.

3.	Leiner DJ. SoSci Survey. Published online 2019.

4.	Lidzba K, Christiansen H, Drechsler R. Conners-3. Conners Skalen zu Aufmerksamkeit und Verhalten – 3. Deutschsprachige Adaptation der Conners 3rd EditionTM (Conners 3TM) von C. Keith Conners. Published online January 1, 2013.
